# Predictors of referral behaviour and intention amongst physicians in a medical consortium based on the theory of planned behaviour: a cross-sectional study in China

**DOI:** 10.3389/fpubh.2023.1159207

**Published:** 2023-08-16

**Authors:** Dongbao Zhao, Shengliang Chen, Sihui Jin, Lijin Chen, Caiyun Zheng, Xin Wang, Yixiang Huang

**Affiliations:** Department of Health Policy and Management, School of Public Health, Sun Yat-sen University, Guangzhou, China

**Keywords:** patient referral, theory of planned behaviour, structural equation modelling, county medical consortium, China

## Abstract

**Introduction:**

To promote patients’ referral across healthcare institutions and integrated care delivery, we identified predictors of physicians’ behaviour and intention to refer patients in a county medical consortium in China on the basis of the theory of planned behaviour (TPB).

**Methods:**

This census-based cross-sectional study was conducted in Yangxi Hospital Group (YHG). All physicians in county hospitals and township health centres were invited to participate. Structural equation modelling was employed to analyse the relationships between referral intention and behaviour and other TPB variables in the group of whole participants and in sub-groups.

**Results:**

In total, 330 physicians participated in this study. One-third of participants were general practitioners, and half of them were from county hospitals. Referral behaviour of females (*χ*^2^ = 20.372, *p* < 0.001), who had lower education levels (*χ*^2^ = 17.859, *p* = 0.001), lower professional title (*χ*^2^ = 14.963, *p* = 0.005), and lower monthly salary (*χ*^2^ = 33.753, *p* < 0.001) were less frequent than the others. Among them, 116 (35.2%), 108 (32.7%), and 106 (32.1%) respondents reported that they had never referred patients, had referred patients 1–9 times, and had referred patients over 10 times during the past 3 months, respectively. The mean score of referral intention was 4.23/5 (SD = 0.71). In the model with all participants, a stronger referral intention (*β* = 0.218, 95% CI = 0.080–0.356) was associated with more frequent referral behaviour. The subjective norm (*β* = 0.703, 95% CI = 0.590–0.817) was the strongest predictor of physicians’ referral intention, followed by perceived behavioural control (*β* = 0.234, 95% CI = 0.090–0.378). Mediated by referral intention, subjective norms (*β* = 0.153, *p* < 0.01) and perceived behavioural control (*β* = 0.190, *p* < 0.01) had significant indirect effects on physicians’ referral behaviour. The model with participants in county hospitals showed similar results to the model with all participants. Meanwhile, in the model with participants in township health centres, there were no significant associations between referral behaviour and other TPB constructs.

**Conclusion:**

Physicians’ referral behaviour was influenced by intention, subjective norms, and perceived behavioural control in Chinese county hospitals.

## 1. Introduction

Referral is defined as a request made to healthcare professionals or organisations for assistance, or an action of primary care physicians who send patients to qualified physicians for a second opinion or therapy when they are unable or unwilling to treat their patients (1). The World Health Organization (WHO) proposed that a referral is an essential part of a comprehensive healthcare system (2). Referrals not only affect the process of patients’ diagnosis and treatment, but also affect their clinical outcomes and expenses (3–5).

The Deepening Health Reform in China, which was jointly launched by the World Bank, WHO, and the Chinese government in 2016, highly emphasised the importance of shaping tiered healthcare systems and promoting patient referrals (6). In rural counties in China, the three-tier healthcare system comprises village clinics, township health centres (THCs), and county hospitals (7). THCs and village clinics provide primary care for the population and serve as gatekeepers. The relatively integrated three-tier county healthcare network in China’s counties functioned well in the planned economy (1949–1992). However, over the past three decades, with the development of a market economy, decentralisation, and the reduction in financial allocations from the central government, this network has been dismantled (8). This resulted in patients seeking affordable healthcare at any facility, without a referral from the gatekeeper (7). Meanwhile, hospitals have amassed more healthcare resources and patient visits than primary care institutions, particularly tertiary hospitals, owing to increased revenue from patients, public finance, and medical insurance funds. From 2009 to 2021, it was estimated that the average annual increase rate of outpatient visits in primary care institutions was 3.44%, whereas that in hospitals was 5.97%. In 2021, the average cost of outpatient visits in rural THCs was $11.12 USD, which was one-fourth of the average cost in hospitals ($45.63 USD). Therefore, the fragmented three-tier network, without referral in rural counties, partially contributed to the high increase of total healthcare expenditures in China.

Since 2009, the tiered referral system in China has been rebuilding to establish high-quality and value-based service delivery. Referrals between different levels of healthcare facilities have increased, especially since 2015 (8). In 2019, The number of two-way referrals was 24.63 million with 17.39 upward referrals (from lower-level facilities to higher-level facilities) and 7.24 downward referrals (from higher-level facilities to lower-level facilities), accounting for 0.29% of the total outpatient visits in China (9, 10). However, compared with the general referral rate of 10–30% in other developed and developing countries, the referral rate in China is quite low (11–14).

Numerous studies have been conducted on the clinical and non-clinical factors that influence referral in China and overseas (15–24). The clinical characteristics of the disease are essential conditions for referral (20, 25–28). Patients diagnosed with severe diseases are referred more frequently, worldwide (20, 25–28). In terms of non-clinical characteristics, a qualitative study suggested that patient and family factors (e.g., patients’ willingness), physician factors (e.g., physicians’ judgement of clinical characteristics), and practice factors (e.g., peer pressure, local practice pattern, and practice environment) are the three main factors influencing family physicians’ referral decision making (22). Regarding physician factors, existing studies revealed that a high education level, comprehensive knowledge about the referral process, and good professional networking with other organisations were facilitators of physicians’ referral behaviour (16–18). In addition to the influence of socio-demographic characteristics on physicians’ referral behaviour, several studies have emphasised the influence of psychological factors as well. Interpersonal factors (e.g., quality of communication with other physicians) (16), social norms (24, 29), and perceived self-efficacy (e.g., success in referrals) (15, 30) can affect physicians’ referral behaviour.

The theory of planned behaviour (TPB) is used to understand and predict behaviours, indicating that behaviours are immediately determined by behavioural intentions and perceived behavioural control, and behavioural intentions are determined by attitudes towards behaviour, subjective norms (SN), and perceived behavioural controls (PBC) (31). Godin et al. recommended applying TPB to predict behaviours and intentions amongst healthcare professionals (32). Several studies have examined the associations of attitude, SN, and PBC with physicians’ intention to refer patients (19, 23, 33). A study on physicians’ intentions to refer cancer patients for psychosocial therapy found that the most important predictors of physicians’ intentions were attitude and SN (19). O’Connell et al. identified SN as the strongest predictor of physicians’ intention to refer children and families to paediatric psychologists (23). However, there is a research gap to explore and compare the effects of attitude, SN, and PBC on referral behaviour of physicians in multi-level hospitals.

In 2017, the Health Commission of China introduced a medical consortium as a new approach to people-centred integrated care (34). By December 2019, 1,408 and 3,346 medical consortia had developed in urban districts and rural counties in China, respectively (35). In the medical consortia, some strategies were adopted to promote referrals, including priority treatment and standard guidelines for referring patients between facilities in the consortia (36). We aimed to identify predictors of physicians’ behaviour and intention to refer patients in a medical consortium on the basis of the TPB to promote patients’ referral across healthcare institutions and integrated care delivery.

## 2. Theoretical model and hypotheses

**Figure 1:**
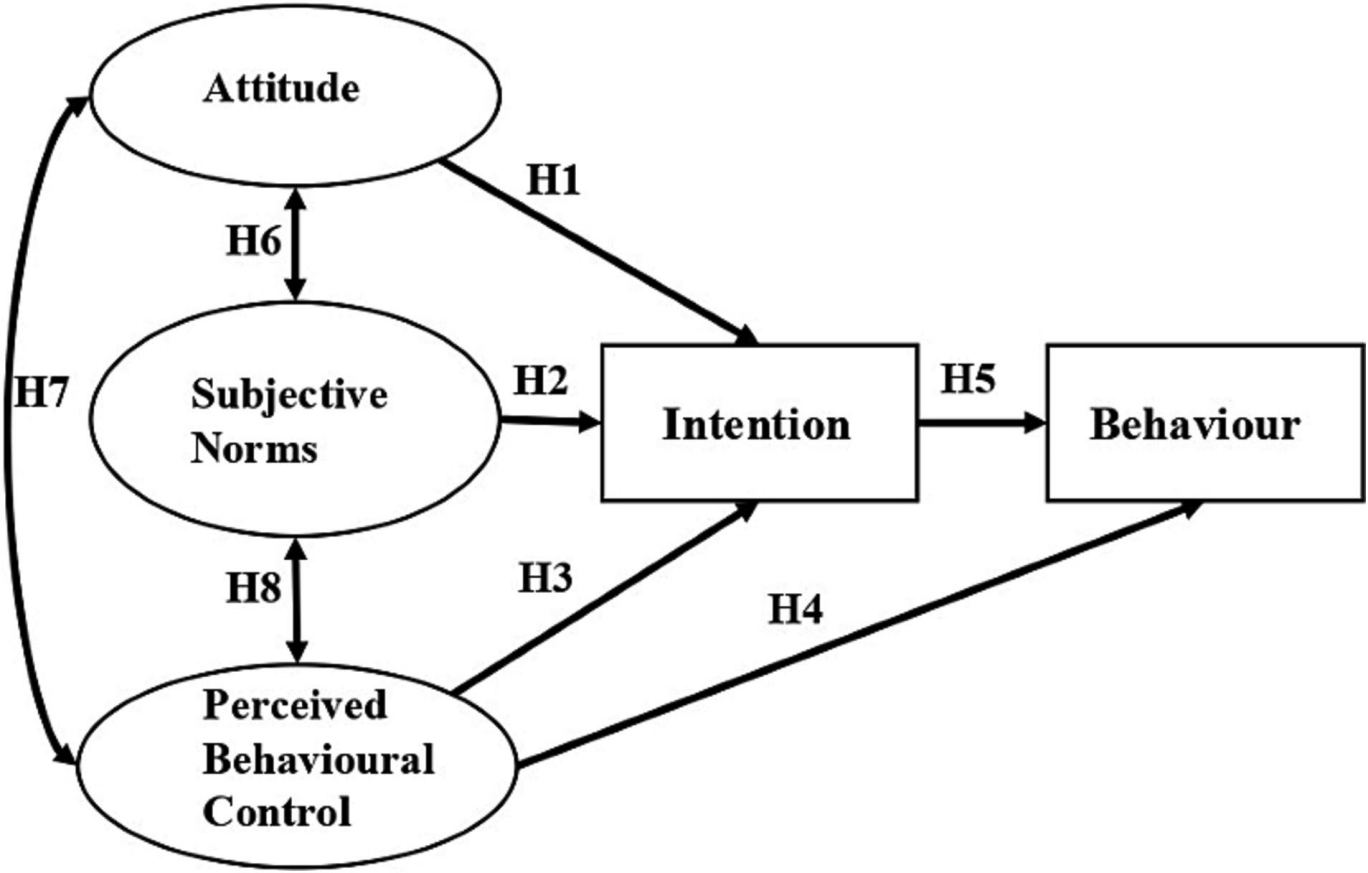
Theoretical model of the study.

TPB was recommended as an appropriate theory to predict health workers’ behaviour (32, 33). The theoretical framework of this study (see Figure 1) was adapted from the TPB model, which predicts that attitudes towards the behaviour, subjective norms, and perceived behavioural controls affect the intention to perform the behaviour (31). According to Ajzen, attitudes towards referrals were defined as whether a physician was in favour of referring patients to other care providers (31). Subjective norms were defined as the perceived social pressure from peers by a physician regarding referrals. Perceived behavioural controls of referral behaviour measured physicians’ perceptions of the ease and difficulty of performing referral behaviours. The referral intention to refer patients measured the degree to which a physician planned to refer the patients. Referral behaviour measured how often a physician referred patients to other healthcare facilities. On the basis of the theoretical framework and literature review, we formulated the following eight hypotheses:

*H1:* Attitudes towards referrals predict intention to refer patients.

*H2:* Subjective norms about referral behaviour predict intention to refer patients.

*H3:* Perceived behavioural control of referral behaviour predict intention to refer patients.

*H4:* Perceived behavioural control of referral behaviour affects referral behaviour.

*H5:* Referral intention affects referral behaviour.

*H6:* Attitude and subjective norms are associated with each other.

*H7:* Attitude and perceived behavioural control are associated with each other.

*H8:* Subjective norms and perceived behavioural control are associated with each other.

## 3. Methods

This is a census-based cross-sectional study targeting physicians in a county hospital group. We reported the study based on STROBE Statement (37).

### 3.1. Study setting

This study was conducted in Yangxi County, Guangdong Province, China in November 2020. Supplementary Table S7 provided additional information on the socio-demographic characteristics of Yangxi County in 2020 (38–40). To rebuild the three-tier integrated county healthcare system, the Yangxi Hospital Group (YHG) was established in 2017. The YHG comprises three county hospitals, eight THCs, and 220 village clinics. To promote integrated healthcare delivery in the YHG, one adopted strategy was strengthening two-way referral between county hospitals and THCs. Primary care in THCs and village clinics was the first point of contact. If necessary, physicians in THCs should refer patients to county hospitals for diagnosis or hospitalisation. Once patients are discharged from county hospitals, they can be referred to THCs for follow-up treatment or rehabilitation. It was estimated that the number of upward referrals and downward referrals within the YHG were 3,911 and 7,517 in 2017–2019, accounting for 0.19 and 0.32% of visits in THCs and county hospitals, respectively, much lower than the expectations of policymakers in the YHG (9, 10).

### 3.2. Sampling and participants

The sample size was 5 or 10 times as large as the number of free parameters in structural equation modelling (SEM) (41, 42). There were 30 free parameters in this study, equal to the number of paths between variables and between observed variables and their errors. The sample size of this study should not be less than 150. In this study, all 121 general practitioners and 290 specialists in the three county hospitals and eight THCs in the YHG were invited to participate in the web survey. The exclusion criteria were (1) work experience of less than 3 months and (2) a leave of absence from work. Participation in the survey was voluntary, with a consent form, and the participants were not compensated. This study complied with the recommendations of the Declaration of Helsinki and was approved by the Ethics Committee of the School of Public Health at Sun Yat-sen University.

### 3.3. Data collection

A web survey was conducted in November 2020. Data were collected using the online data collection tool ‘SO JUMP’ (43). An online questionnaire was sent to all potential participants’ mailboxes through an automated office system of the YHG. The questionnaire comprises two sections. The first section included socio-demographic characteristics of participants, including gender, age, organisation, type of profession, marital status, education level, years of practice, professional title, and monthly salary. The second section measured the TPB variables (attitudes, subjective norms, perceived behavioural controls, intention, and behaviour related to patient referral).

Supplementary Table S1 shows the items and ratings used to measure TPB variables. Referral behaviour, as an outcome variable, was prospectively measured using a three-point Likert scale, which was classified by the number of referrals during the past 3 months (1 = zero, 2 = 1–9 times, 3 = over 10 times). Referral intention was rated on a five-point Likert scale, ranging from 1 (very unlikely) to 5 (very likely). The variables of attitude, subjective norms, and perceived behavioural control were measured using 10 items derived from the TPB scale (34). Each item on attitude and perceived behavioural control was rated on a three-point Likert scale ranging from 1 (disagree) to 3 (agree). Each item of the subjective norms was rated on a five-point Likert scale ranging from 1 (strongly disagree) to 5 (strongly agree).

### 3.4. Data analysis

Descriptive analyses were performed to illustrate the participants’ demographic characteristics and ratings of the TPB constructs. Chi square test and Fisher exact test were employed to compare the characteristics of physicians with different referral behaviours. Participants were divided into two groups regarding their institutions: Group 1 with participants in county hospitals and Group 2 with participants in THCs. Wilcoxon rank-sum test was used to examine the difference in measurement scores of TPB constructs between the two groups.

SEM was employed to verify the proposed hypotheses. This was conducted on the basis of the two-step approach recommended by Anderson (44). In the first step, a measurement model was established to examine the relationships between latent variables (attitude, subjective norms, and perceived behavioural control) and their indicators. The indicator is valid if the factor loading on its latent variable exceeds 0.5 (45). To validate the questionnaire further, the reliability, convergent validity, and discriminant validity of each construct were calculated. Reliability was assessed using Cronbach’s *α* (>0.6) (44). Convergent validity was assessed using the average variance extracted (AVE > 0.36) and composite reliability (CR > 0.6) for all constructs (20). Discriminant validity was tested using the Fornell–Larcker criterion. When the average shared variance of each construct and its diagonal values exceeded the shared variance with the other constructs, the Fornell–Larcker criterion was met. The square roots of the AVE of each construct should be higher than the correlations with other constructs (46). Constructs that failed to achieve reliability and validity were adjusted by deleting poor factor-loading indicators. Resultantly, the final measurement model adopted for structural analysis was obtained.

In the second step, a structured equation model was constructed to analyse the standardised path coefficients. Referral intention and behaviour were added to the final measurement model as the observed variables. Correlations within the variables were modified according to the modification index (MI) into a well-fitting model. The fitness of the data in the SEM model was assessed using several recommended criteria: the comparative fit index (CFI > 0.95), Tucker–Lewis index (TLI > 0.95), root-mean-square error of approximation (RMSEA < 0.06), and standardised root-mean-square residual (SRMR < 0.06) (47–50). According to Cohen (51), if all direct effects are significant at the same significant level (*α*), the indirect effects derived from their product will be significant at the same level. In the single mediator model, indirect effects of attitude, subjective norm and perceived behavioural control on referral behaviour were mediated by referral intention. In total, 95% confidence intervals (CIs) and standard errors (SEs) of the standardised path coefficients (*β*) in the model were calculated. Statistical analyses were performed using STATA (version 15.0). A *p* value <0.05 was considered statistically significant.

### 3.5. Sensitivity analysis

A nominal range sensitivity analysis was conducted to evaluate the effect of varied classifications of referral behaviour on the results. As nominal sensitivity analysis addressed only a potential small portion of the possible space of input values (52), nine extra classifications were chosen to build extra models (Model 1 to 9) for the three structural equation models (See Supplementary Table S3). The significance and values of coefficients were compared (53), and the changes of CFI (ΔCFI < 0.01) and RMSEA (ΔRMSEA < 0.015) between the extra models and original model were calculated to indicate the differences (54). If insignificant differences were found between the original model and the majority of extra models, the original model was considered insensitive to the classification (0, 1–9, over 10). The results showed that the models were not sensitive to the classification of referral behaviour with ΔCFI < 0.01 and ΔRMSEA < 0.015 (See Supplementary Tables S4–S6).

## 4. Results

### 4.1. Participant characteristics

A total of 330 physicians provided written informed consent and completed an online questionnaire, with a response rate of 80.30%. The socio-demographic characteristics of the participants are presented in Table 1. Amongst the participants, 59.1% were male, 49.1% were from county hospitals, 42.7% had a bachelor’s degree, 58.2% had over 10 years of practice experience, and approximately half had a monthly salary of less than ¥5,000 ($689.95 USD). In terms of practice type, two-thirds of the participants were specialists, and one-third were general practitioners. Participants reported that 116 of them had never referred patients, 108 had referred patients 1–9 times, and 106 had referred patients more than 10 times in the past 3 months, accounting for 35.2, 32.7, and 32.1% of the total participants, respectively. Referral behaviour of females (*χ*^2^ = 20.372, *p* < 0.001), who had lower education levels (*χ*^2^ = 17.859, *p* = 0.001), lower professional title (*χ*^2^ = 14.963, *p* = 0.005), and lower monthly salary (*χ*^2^ = 33.753, *p* < 0.001) were less frequent than the others.

**Table 1:** Demographic characteristics of the participants (*n* = 330).

Characteristic	Referral number = 0 (*n* = 116, 35.2%)		Referral number = 1–9 (*n* = 108, 32.7%)		Referral number over 10 (*n* = 106, 32.1%)		*χ*2/*F*	*p*	Total (*n* = 330)	
	*N*	%	*N*	%	*N*	%			*N*	%
Gender							20.372	<0.001***		
Male	51	26.2	66	33.8	78	40.0			195	59.1
Female	65	48.1	42	31.1	28	20.7			135	40.9
Age, years							2.744	0.840		
20–29	32	41.6	24	31.2	21	27.3			77	23.3
30–39	40	33.1	43	35.5	38	31.4			121	36.7
40–49	34	33.3	32	31.4	36	35.3			102	30.7
50 and over	10	33.3	9	30.0	11	36.7			30	9.1
Organisations							1.380	0.502		
County hospital	54	33.3	51	31.5	57	35.2			162	49.1
Township health centre	62	36.9	57	33.9	49	29.2			168	50.9
Type of profession							7.915	0.019		
General practitioner	27	24.8	43	39.4	39	35.8			109	33.0
Specialist	89	40.3	65	29.4	67	30.3			221	67.0
Marital status							6.504	0.150		
Unmarried	27	45.0	20	33.3	13	21.7			60	18.2
Married	85	32.4	85	32.4	92	35.1			262	79.4
Others	4	50.0	3	37.5	1	12.5			8	2.4
Education level							17.859	0.001**		
Secondary school or less	11	52.4	6	28.6	4	19.0			21	6.4
Junior college	73	43.5	46	27.4	49	29.2			168	50.9
Bachelor or above	32	22.7	56	39.7	53	37.6			141	42.7
Years of practice							4.445	0.349		
<5 years	33	42.9	24	31.2	20	26.0			77	23.3
5–10 years	19	31.1	24	39.3	18	29.5			61	18.5
>10 years	64	33.3	60	31.3	68	35.4			192	58.2
Professional title							14.963	0.005**		
Junior or less	91	41.9	66	30.4	60	27.6			217	65.8
Intermediate	18	25.0	24	33.3	30	41.7			72	21.8
Senior	7	17.1	18	43.9	16	39.0			41	12.4
Monthly salary (USD)							33.753	<0.001***		
<¥5,000 ($689.95)	79	44.9	61	34.7	36	20.5			176	53.3
¥5,000–¥8,000 ($689.95–$1,105.15)	32	31.4	27	26.5	43	42.2			102	30.9
>¥8,000 ($1,105.15)	5	9.6	20	38.5	27	51.9			52	15.8

*p < 0.05, **p < 0.01, and ***p < 0.001.

### 4.2. Measurement scores

As shown in Table 2, the attitudes of the respondents indicated a preference for referring patients (mean = 2.40, SD = 0.75). Respondents reported a high subjective norm score (mean = 4.21, SD = 0.74), strong perceived behavioural control (mean = 2.46, SD = 0.65), and high referral intention (mean = 4.23, SD = 0.71) regarding referring patients. Participants in county hospitals (Group 1) suggested higher scores of all indicators of attitude (*z* = 3.777–6.181, *p* > 0.001) and a lower score of SN3 (*z* = −2.53, *p* = 0.012) compared with those in THCs (Group 2).

**Table 2:** Measurement scores of TPB constructs.

Construct	Group 1		Group 2		Rank sum test		Total	
	Mean	SD	Mean	SD	*z*	*p*	Mean	SD
Attitude	2.60	0.67	2.20	0.77	8.810	<0.001***	2.4	0.75
ATT1	2.65	0.65	2.33	0.83	3.777	<0.001***	2.49	0.77
ATT2	2.79	0.47	2.41	0.64	6.181	<0.001***	2.59	0.59
ATT3	2.35	0.77	1.87	0.71	5.802	<0.001***	2.11	0.77
Subjective Norm	4.16	0.78	4.26	0.69	−1.87	0.061	4.21	0.74
SN1	4.22	0.75	4.30	0.69	−0.95	0.342	4.26	0.72
SN2	4.33	0.64	4.32	0.69	0.105	0.917	4.32	0.66
SN3	3.92	0.87	4.16	0.70	−2.53	0.012*	4.04	0.80
Perceived Behavioural Control	2.47	0.63	2.44	0.66	0.786	0.432	2.46	0.65
PBC1	2.65	0.57	2.59	0.59	1.130	0.259	2.62	0.58
PBC2	2.61	0.58	2.60	0.58	0.193	0.847	2.60	0.58
PBC3	2.49	0.61	2.46	0.67	0.147	0.883	2.47	0.64
PBC4	2.15	0.64	2.12	0.67	0.358	0.720	2.13	0.66
Referral Intention	4.26	0.74	4.21	0.68	0.942	0.346	4.23	0.71

The scores of attitude, subjective norm and perceived behavioural control were substituted by average values of their indicators. SD, standard deviation. *p < 0.05, **p < 0.01, and ***p < 0.001.

### 4.3. Measurement model

The reliability and convergent validity of the three measurement models (Tables 3, 4) were calculated. All factor loadings were above 0.5, which is acceptable for SEM. In the model with all participants (Table 3), Cronbach’s *α* (>0.6), CR scores (>0.6), and AVEs (>0.36) were appropriate for each construct after adjustment of omitting PBC4. In the initial model of Group 1, each Cronbach’s *α* and CR were above 0.6 and AVE was above 0.36. As 3–5 indicators were recommended by Bagozzi and Baumgartner as the best (55), it was not necessary to omit indicators of attitude to get better convergent validity in the model of Group 2. However, a relatively large adjustment was made to get acceptable Cronbach’s *α*, CR, AVE and discriminant validity. ATT2, ATT3, PBC1, and PBC3 were omitted in the final model, and Cronbach’s *α* (>0.6), CR (>0.6), and AVE (>0.36) were acceptable. As shown in Table 5, the value of the square root of the AVE for each construct was greater than the correlation between each construct with any other construct.

**Table 3:** Reliability and convergent validity of the measurement model of total participants (*n* = 330).

Construct/Indicators	Initial model				Final model			
	Factor loading	Cronbach’s *α*	CR	AVE	Factor loading	Cronbach’s *α*	CR	AVE
Attitude		0.768	0.769	0.529		0.767	0.772	0.534
ATT1	0.834				0.843			
ATT2	0.651				0.653			
ATT3	0.685				0.681			
Subjective Norms		0.767	0.788	0.559		0.767	0.788	0.559
SN1	0.709				0.708			
SN2	0.884				0.886			
SN3	0.626				0.624			
Perceived Behavioural Control		0.775	0.781	0.474		0.739	0.756	0.511
PBC1	0.654				0.661			
PBC2	0.803				0.834			
PBC3	0.666				0.634			
PBC4	0.616				omitted			

AVE, average variance extracted; CR, composite reliability. The initial model is a three-factor model with all indicators. The final model is the model adopted for structural analysis with all indicators except PBC4.

**Table 4:** Reliability and convergent validity of the measurement models of Group 1 (*n* = 162) and Group 2 (*n* = 168).

Construct/Indicators	Group 1 (*n* = 162)				Group 2 (*n* = 168)							
	Initial Model (Final Model)				Initial Model				Final Model			
	Factor loading	Cronbach’s *α*	CR	AVE	Factor loading	Cronbach’s *α*	CR	AVE	Factor loading	Cronbach’s *α*	CR	AVE
Attitude		0.705	0.727	0.475		0.764	0.757	0.513		-	0.974	0.974
ATT1	0.875				0.819				0.998			
ATT2	0.533				0.669				omitted			
ATT3	0.623				0.681				omitted			
Subjective Norms		0.757	0.752	0.504		0.783	0.788	0.555		0.783	0.787	0.552
SN1	0.752				0.707				0.706			
SN2	0.878				0.808				0.806			
SN3	0.632				0.71				0.714			
Perceived Behavioural Control		0.805	0.808	0.519		0.746	0.749	0.43		0.626	0.628	0.458
PBC1	0.768				0.592				omitted			
PBC2	0.868				0.737				0.722			
PBC3	0.638				0.654				omitted			
PBC4	0.569				0.637				0.629			

AVE, average variance extracted; CR, composite reliability.

**Table 5:** Discriminant validity of the final measurement model.

Model	Construct	Attitude	Subjective Norm	Perceived Behavioural Control
Group 1	Attitude	**0.689**		
(*n* = 162)	Subjective Norms	0.607	**0.710**	
	Perceived Behavioural Control	0.456	0.618	**0.720**
Group 2	Attitude	**0.987**		
(*n* = 162)	Subjective Norms	0.387	**0.743**	
	Perceived Behavioural Control	0.672	0.660	**0.677**
Total	Attitude	**0.731**		
(*n* = 162)	Subjective Norms	0.442	**0.748**	
	Perceived Behavioural Control	0.665	0.506	**0.715**

Values that are in bold are the square roots of AVE, and the off diagonals are correlations. AVE, average variance extracted.

### 4.4. Structural model and hypotheses test

**Figure 2:**
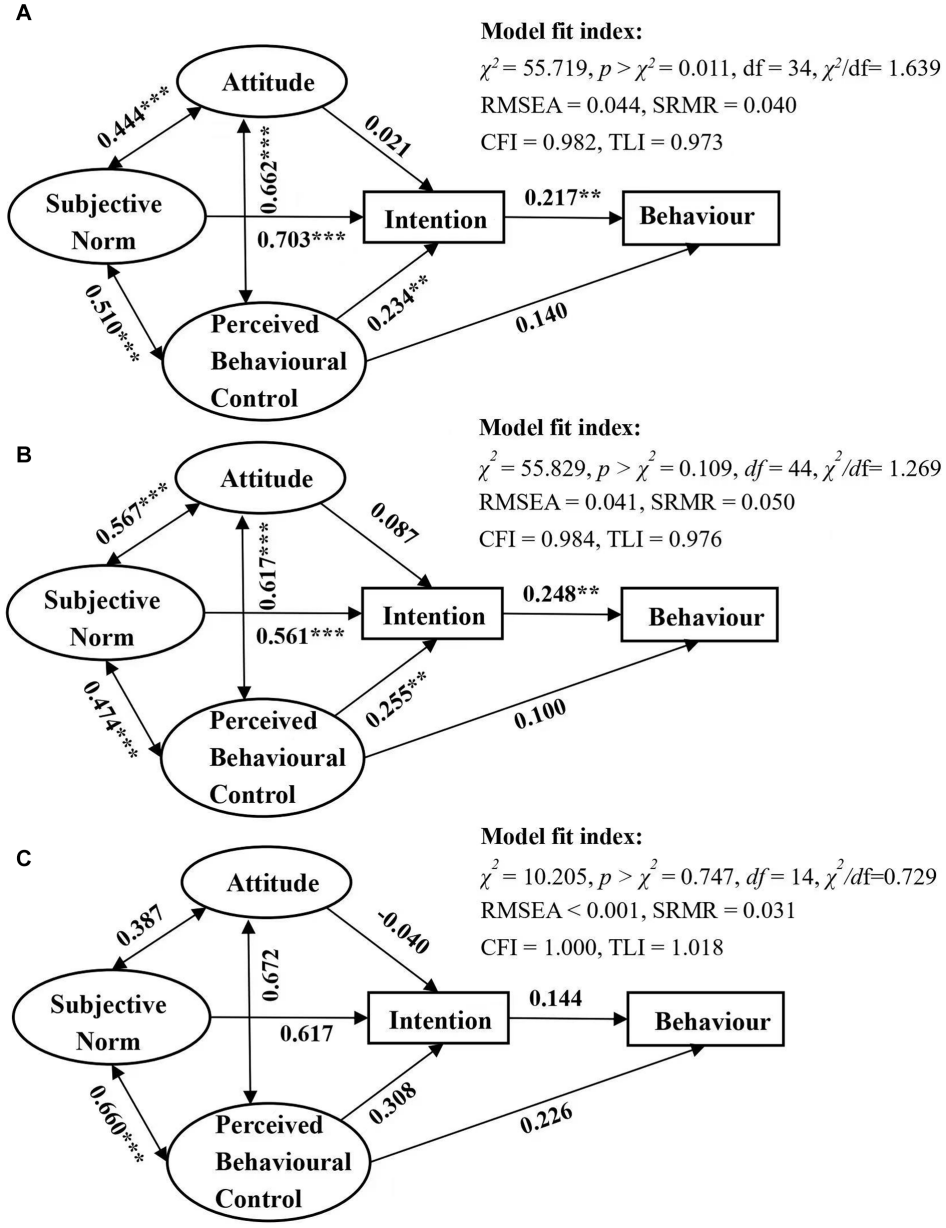
Structure equation model based on the theory of planned behaviour. Standardized path coefficients are presented. **(A)** Model of total participants (*n* = 330), **(B)** model of participants in county hospitals (Group 1: *n* = 162), and **(C)** model of participants in THCs (Group 2: *n* = 168). **p < 0.01 and ***p < 0.001.

Standardized path coefficients of the three structural models were presented in Figure 2 and Supplementary Table S2. In the model with all participants, standardised parameter estimates indicated that a stronger referral intention (Total: *β* = 0.217, 95% CI = 0.079, 0.356) was associated with more frequent referral behaviour. The subjective norms were the strongest predictor of physicians’ referral intention (Total: *β* = 0.703, 95% CI = 0.590, 0.817), followed by perceived behavioural control (Total: *β* = 0.234, 95% CI = 0.090, 0.378). The associations amongst attitude, subjective norms, and perceived behavioural control were significant. Contrary to our hypothesis, the effects of attitude on referral intention (Total: *β* = 0.021, 95% CI = −0.111, 0.154) and perceived behavioural control on referral behaviour (Total: *β* = 0.140, 95% CI = −0.015, 0.295) were not statistically significant. Therefore, H2, H3, and H5–H8 are supported by the results (see Supplementary Table S2). The final structural model reached a very good fit with RMSEA = 0.044 (<0.06), CFI = 0.982 (>0.95), TLI = 0.973 (>0.95), and SRMR = 0.040 (<0.06). Similarly, in the model of Group 1, the effect of subjective norm (Group 1: *β* = 0.561, 95% CI = 0.425, 0.697) and perceived behavioural control (Group 1: *β* = 0.255, 95% CI = 0.092, 0.418) on referral intention and the effect of intention (Group 1: *β* = 0.248, 95% CI = 0.065, 0.429) on referral behaviour were significant. The model of Group 1 also reached a good fit (RMSEA = 0.041 < 0.06, SRMR = 0.050 < 0.06, CFI = 0.984 > 0.95, TLI = 0.976 > 0.95). However, no significant path coefficient was found in the model of Group 2 except covariance between subjective norm and perceived behavioural control (H8).

### 4.5. Indirect and total effects

We also tested the indirect and total effects of the TPB variables on referral behaviour. Non-standardised coefficients (with 95% CI) and standardised coefficients were shown in Table 6. In models with all participants and Group 1, subjective norm (Total: *β* = 0.153, *p* < 0.01; Group 1: *β* = 0.138, *p* < 0.01) and perceived behavioural control (Total: *β* = 0.509, *p* < 0.05; Group 1: *β* = 0.063, *p* < 0.05) had significant indirect effects on physicians’ referral behaviour, whereas attitude’s indirect effects on physicians’ referral behaviour were not significant (Total: *β* = 0.005, *p* > 0.05; Group 1: *β* = 0.022, *p* > 0.05). Referral intention had the largest total effect on referral behaviour (Total: *β* = 0.217, *p* < 0.01; Group 1: *β* = 0.248, *p* < 0.01), followed by perceived behavioural control (Total: *β* = 0.190, *p* < 0.01), and subjective norm (Total: *β* = 0.153, *p* < 0.01) in the model with all participants and by subjective norm (Group 1: *β* = 0.138, *p* < 0.01) in the model of Group 1. Though the indirect effect of perceived behavioural control on referral behaviour was found in the model of Group 1, the direct and total effect (Group 1: *β* = 0.162, *p* > 0.01) were insignificant. There was so significant association between referral behaviour and other TPB constructs in the model of Group 2.

**Table 6:** Non-standardised and standardised effect size values.

Relationship	Total model (*n* = 330)			Group 1 (*n* = 162)			Group 2 (*n* = 168)		
	NS-eff	95%CI	*β*	NS-eff	95%CI	*β*	NS-eff	95%CI	*β*
Direct effect									
Intention ←									
Attitude	0.024	(−0.125, 0.172)	0.021	0.113	(−0.121, 0.347)	0.087	−0.033	(−149.262, 149.196)	−0.040
SN	1.000	–	0.703***	1.000	–	0.561***	1.000	–	0.617
PBC	0.410	(0.155, 0.665)	0.234**	0.415	(0.150, 0.679)	0.255**	0.496	(−215.061, 216.054)	0.308
Behaviour ←									
PBC	0.282	(−0.033, 0.597)	0.140	0.181	(−0.181, 0.543)	0.100	0.433	(−0.081, 0.947)	0.226
Intention	0.251	(0.089, 0.413)	0.217**	0.275	(0.068, 0.482)	0.248**	0.172	(−0.108, 0.451)	0.144
Indirect effect									
Behaviour ←									
Attitude	0.006	(−0.031, 0.043)	0.005	0.031	(−0.037, 0.100)	0.022	−0.006	(−25.615, 25.603)	−0.006
SN	0.251	(0.089, 0.413)	0.153**	0.275	(0.068, 0.482)	0.138**	0.172	(−0.108, 0.451)	0.089
PBC	0.103	(0.011, 0.194)	0.509*	0.114	(0.002, 0.226)	0.063*	0.084	(−36.906, 37.077)	0.044
Total effect									
Intention ←									
Attitude	0.024	(−0.125, 0.172)	0.021	0.113	(−0.121, 0.347)	0.087	−0.033	(−149.262, 149.196)	−0.040
SN	1.000	–	0.703***	1.000	–	0.561***	1.000	–	0.617
PBC	0.410	(0.155, 0.665)	0.234**	0.415	(0.150, 0.679)	0.255**	0.496	(−215.061, 216.054)	0.308
Behaviour ←									
Attitude	0.006	(−0.031, 0.043)	0.005	0.031	(−0.037, 0.100)	0.022	−0.006	(−25.615, 25.603)	−0.006
SN	0.251	(0.089, 0.413)	0.153**	0.275	(0.068, 0.482)	0.138**	0.172	(−0.108, 0.451)	0.089
PBC	0.385	(0.102, 0.667)	0.190**	0.295	(−0.030, 0.619)	0.162	0.518	(−36.476, 37.512)	0.270
Intention	0.251	(0.089, 0.413)	0.217**	0.275	(0.068, 0.482)	0.248**	0.172	(−0.108, 0.451)	0.144

Three models were built and modified separately. NS-eff, non-standardised effects; *β*, standardised effects; CI, confidence interval; SN, subjective norm; PBC, perceived behavioural control. *p < 0.05, **p < 0.01, and ***p < 0.001.

## 5. Discussion

### 5.1. Main findings

To bridge the knowledge gap regarding physicians’ actual referral behaviour and its influencing factors in county healthcare systems in China, this study verified the theoretical model on the basis of the TPB. The SEM approach was used to simultaneously investigate the factors influencing physicians’ referral behaviour and the interactions between these factors. We found that the majority of physicians reported intentions (4.23/5) to refer patients. However, more than one-third of all participants did not refer patients in the past 3 months. Referral behaviour of female, who had lower education levels, lower professional title and lower monthly salary were less frequent. Physicians in THCs reported less positive attitude compared with those in county hospitals. Physicians’ referral behaviour depended on their intentions, which were directly influenced by subjective norms and perceived behavioural control. Therefore, subjective norms and perceived behavioural control significantly affected physicians’ referral behaviour, mediated by referral intention. All but two hypotheses (H1 and H4) in the model were supported, confirming that TPB was an acceptable theoretical foundation for this study. However, the findings were applicable for participants in county hospitals and were not applicable for participants in THCs.

### 5.2. Comparison with other studies

Consistent with the TPB and previous studies, subjective norms and perceived behavioural control were important predictors of physicians’ intentions and behaviours related to patient referral, antibiotic prescriptions, and adoption technology. A study in the UK found that subjective norms played a vital role in determining healthcare professionals’ intention to refer children with physical illnesses to paediatric psychologists (23). According to Wang’s study in tertiary hospitals in China, stronger subjective norms and perceived behavioural control were associated with a higher intention to refer patients to lower-level hospitals (33).

The attitudes towards patients’ referral did not significantly affect physicians’ intentions or behaviours. This finding is not in line with those of previous studies. A study in the US concluded that the most significant predictors of the intention to refer cancer patients for psychosocial support were attitude and subjective norms (19). However, according to a previous study, the efficacy of TPB’s predictive value can vary depending on the context, behaviour, and characteristics of the population performing the behaviour (56). Our study was conducted in county health systems, targeting physicians in county hospitals and THCs of a medical consortium. Patients for referral were not diagnosed with certain diseases. Therefore, with different findings regarding physicians’ attitudes towards patient referral, this study provides additional evidence to illustrate the importance of intention, subjective norms, and perceived behavioural control in shaping physicians’ referral behaviour in Chinese county medical consortia.

In our study, the largest regression coefficient was in the path from subjective norms to intention. We measured subjective norms from the perspectives of leaders, colleagues, and patients. The effects of subjective norms from leaders and colleagues on referral intention have also been confirmed in some previous studies (19, 57). The support of leaders was vital in establishing collaborative relationships amongst institutions and in increasing physicians’ awareness of referrals (58). Regarding the patients’ interest in referrals, Armstrong noted that general practitioners with a high referral rate were more likely to report pressure from patients (59). O’Connell found that the interests of patients’ families were the most significant determinants of physicians’ referral intention (23). Similarly, in our study, physicians’ referral intentions can be affected by patients’ interests. Contrastingly, Wang reported a non-significant effect of patients’ interests on physicians’ referral behaviour amongst several subjective norms in China (57). A possible explanation could be that patients are more likely to follow physicians’ advice in Chinese urban areas than in rural areas due to the high levels of education and health literacy (60).

In this study, the SEM showed that perceived behavioural control indirectly affected referral behaviour, mediated through referral intention, having no direct effect on referral behaviour. Factors shaping perceived behavioural control of physicians’ referrals included the referral system, referral process, and mutual recognition of test results. We found that a reasonable referral process and specified referral system can improve referral intentions. Consistent with our findings, previous studies in China also found that ambiguous referral criteria and cumbersome referral processes were common barriers to referrals (61, 33). The mutual recognition of patients’ test results implies that these results are shared in an interconnected information system across institutions. If necessary, physicians in different institutions can access the results and recognise their accuracy. A possible explanation for the association between mutual recognition of test results and referral behaviour might be attributed to the decrease in cost for repeated tests. In a web survey of 551 patients from 30 provinces of China, 70.96% (369/520) of the patients reported a decrease in cost and a simplification in the process due to mutual recognition of test results (62).

No effects of attitude, subjective norm, perceived behavioural control, and intention on referral behaviour of physicians in THCs were inconsistent with previous studies. In previous studies, lack of information sharing (61), unclear criteria for referral (63), complicated formalities (2), and patients’ willingness (64) restricted upward referral behaviours of physicians in THCs. This might partially result from different collaboration relationships between county hospitals and THCs. In theory, county hospitals and THCs in medical consortia have a collaborative relationship of referral, while county hospitals and THCs not in medical consortia might not have the relationship.

### 5.3. Policy implications

Strategies to strengthen subjective norms regarding referrals should focus on developing a favourable culture for patient referral. On the one hand, sharing and disclosure of information related to referrals are alternatives to support and supervise referrals of patients (65). An important approach to strengthen subjective norms is to enhance the perceived pressure from leaders and colleagues. Sharing information related to referrals could promote sharing of experiences and opinions among physicians. Meanwhile, disclosure of the information related to referral-based performance evaluation by institutions could virtually convey the leaders’ views on referral to physicians and promote inter-group competitions. On the other hand, considering the lack of compulsory gatekeepers and a patient’s freedom to choose medical institutions in China, substantial effort is required to increase patients’ referral willingness. One of the most widely reported predictors of patients’ downward referral interest is the treatment effect of primary healthcare institutions (2). Participants who evaluated a better treatment effect in primary healthcare institutions were more willing to be referred to these institutions. According to a study in Hubei Province, China, the primary healthcare system is qualified as a healthcare gatekeeper for patients, especially for those with noncommunicable diseases (66). Therefore, the managers in medical consortia can first pilot the referral of patients with noncommunicable diseases by promoting their interests in two-way referrals (67).

Considering perceived behavioural control, medical consortia managers should pay attention to the referral system and its operations. On the one hand, detailed and feasible guidelines on referral criteria and related training are essential to increase physicians’ intention to refer patients. On the other hand, referral management systems allowing for information sharing can improve the efficiency of physicians’ referral and significantly improve continuous care delivery for patients (68).

### 5.4. Strengths and limitations

Our study is the first to investigate the factors influencing physicians’ referral intentions and behaviour by applying the TPB to a county medical consortium. However, this study holds some limitations. Firstly, it was a cross-sectional study, which prevented us from making causal associations between intention and behaviour; thus, well-designed prospective studies should be conducted to verify this relationship. Secondly, we did not include factors that are not TPB constructs but have potentially greater effects on physicians’ referral behaviour. For example, lack of self-regulation, an aspect of actual control over behaviour, can reduce the predictive validity of intentions in previous studies (69). Thirdly, 19.71% of the targeted physicians did not respond to this study. With the response rate of 80.29%, we might over-estimate the referral behaviours, referral attitudes and intentions of all physicians. Finally, recall bias may have occurred because the number of referral behaviours was self-reported. In the YHG, physicians can refer patients through online platforms, messages, and calls through their personal phones, which made it impossible for us to trace the actual number. However, as Armitage concluded, when behaviour measures were self-reported, the TPB accounted for over 11% of the variance in behaviour than when behaviour was objectively measured (70). Further studies should collect actual data related to the referral management system.

## 6. Conclusion

A majority of physicians intended to refer patients to the county medical consortium, but only a few referred the patients. We provide supportive evidence that physicians’ referral behaviour in Chinese county hospitals was influenced by intention, subjective norms, and perceived behaviour control instead of attitudes. Developing a favourable culture for information sharing and disclosure related to physicians’ referral behaviour, strengthening the capacity of healthcare service delivery in primary healthcare institutions, developing detailed and feasible guidelines on referral criteria, and improving the referral management system with information sharing can promote physicians’ referral intention and behaviour and enhance integrated care delivery for patients in county medical consortia of China and other developing countries sharing a similar county health system background. It’s necessary to further explore the factors influencing referral behaviour from the perspective of both physicians and institutions.

## Data availability statement

The raw data supporting the conclusions of this article will be made available by the authors, without undue reservation.

## Ethics statement

The studies involving human participants were reviewed and approved by Ethics Committee of the School of Public Health at Sun Yat-sen University (ref 2020 No. 045). The patients/participants provided their written informed consent to participate in this study.

## Author contributions

XW and YH: conceptualization, validation, and resources. XW and DZ: methodology. DZ: software, formal analysis, and writing—original draft. SC, SJ, and LC: investigation. DZ and CZ: data curation. SJ, CZ, and XW: writing—review and editing. All authors contributed to the article and approved the submitted version.

## Funding

This work was supported by the National Social Science Fund of China (grant number 18BGL218) and the National Natural Science Foundation of China (grant number 71804202). The funders had no role in preparation of the manuscript or decision to publish.

## Conflict of interest

The authors declare that the research was conducted in the absence of any commercial or financial relationships that could be construed as a potential conflict of interest.

## Publisher’s note

All claims expressed in this article are solely those of the authors and do not necessarily represent those of their affiliated organizations, or those of the publisher, the editors and the reviewers. Any product that may be evaluated in this article, or claim that may be made by its manufacturer, is not guaranteed or endorsed by the publisher.
