# Supplementary material for: Predictors of referral behaviour and intention amongst physicians in a medical consortium based on the theory of planned behaviour: a cross-sectional study in China
Source: Front Public Health. 2023 Aug 16;11:1159207. doi: 10.3389/fpubh.2023.1159207 (PMC10466405; doi:10.3389/fpubh.2023.1159207)
Supplement: Supplementary file 1 [file Data_Sheet_1.docx]

Supplementary Material

Predictors of referral behaviour and intention of physicians in a medical consortium based on the theory of planned behaviour: A cross-sectional study in China

Dongbao Zhao, Shengliang Chen, Sihui Jin, Lijin Chen, Caiyun Zheng, Xin Wang^*^, Yixiang Huang^*^

*** Correspondence:** Xin Wang**:** wangxin25@mail.sysu.edu.cn, Yixiang Huang: huangyx@mail.sysu.edu.cn

# Supplementary Table

Supplementary Table 1. Description and ranges of TPB variables

| **Construct** | **Description** | **Score range** |
| --- | --- | --- |
| Attitude | |  |
| ATT1* | You agree with the establishment of the referral system. | 1–3 |
| ATT2* | The cooperation amongst physicians would be improved after establishing the referral system. | 1–3 |
| ATT3* | Your income would be improved after establishing the referral system. | 1–3 |
| Subjective Norms | |  |
| SN1 | Managers of the YHG suggest you refer patients. | 1–5 |
| SN2 | Your colleagues and leaders of your organisation suggest you refer patients. | 1–5 |
| SN3 | Most patients and their families suggest you refer patients. | 1–5 |
| Perceived Behavioural Control | |  |
| PBC1* | The criteria for referral are specified. | 1–3 |
| PBC2* | The referral process is perfectly logical and reasonable. | 1–3 |
| PBC3* | The test results were mutually recognised across institutions. | 1–3 |
| PBC4* | There is good cooperation amongst physicians in county-level hospitals and township health centres. | 1–3 |
| Referral Intention | If necessary, how likely are you to refer patients in the next month? | 1–5 |

Supplementary Table 2. Hypothesis testing

| **Model** | **Hypothesis** | **Relationship** | ***β*** | **SE** | **95% CI** | ***p-value*** | **Support** |
| --- | --- | --- | --- | --- | --- | --- | --- |
| Total | H1 | Intention ← Attitude | 0.021 | 0.068 | (-0.111, 0.154) | 0.753 | No |
| (n=330) | H2 | Intention ←SN | 0.703 | 0.058 | (0.590, 0.817) | <0.001 | Yes |
|  | H3 | Intention ←PBC | 0.234 | 0.073 | (0.090, 0.378) | 0.001 | Yes |
|  | H4 | Behaviour ←PBC | 0.140 | 0.079 | (-0.015, 0.295) | 0.078 | No |
|  | H5 | Behaviour ← Intention | 0.217 | 0.071 | (0.079, 0.356) | 0.002 | Yes |
|  | H6 | Cov (Attitude, SN) | 0.444 | 0.057 | (0.332, 0.556) | <0.001 | Yes |
|  | H7 | Cov (Attitude, PBC) | 0.662 | 0.048 | (0.569, 0.756) | <0.001 | Yes |
|  | H8 | Cov (SN, PBC) | 0.510 | 0.057 | (0.399, 0.321) | <0.001 | Yes |
| Group 1 | H1 | Intention ← Attitude | 0.087 | 0.092 | (-0.093, 0.2628) | 0.343 | No |
| (n=162) | H2 | Intention ←SN | 0.561 | 0.069 | (0.425, 0.697) | <0.001 | Yes |
|  | H3 | Intention ←PBC | 0.255 | 0.083 | (0.092, 0.418) | 0.002 | Yes |
|  | H4 | Behaviour ←PBC | 0.100 | 0.101 | (-0.099, 0.298) | 0.325 | No |
|  | H5 | Behaviour ← Intention | 0.247 | 0.093 | (0.065, 0.429) | 0.008 | Yes |
|  | H6 | Cov (Attitude, SN) | 0.568 | 0.069 | (0.432, 0.703) | <0.001 | Yes |
|  | H7 | Cov (Attitude, PBC) | 0.617 | 0.073 | (0.475, 0.759) | <0.001 | Yes |
|  | H8 | Cov (SN, PBC) | 0.474 | 0.075 | (0.326, 0.621) | <0.001 | Yes |
| Group 2 | H1 | Intention ← Attitude | -0.040 | 67.444 | (-132.229, 132.148) | 1.000 | No |
| (n=168) | H2 | Intention ←SN | 0.617 | 9.208 | (-17.430, 18.665) | 0.947 | No |
|  | H3 | Intention ←PBC | 0.308 | 68.280 | (-133.518, 134.134) | 0.996 | No |
|  | H4 | Behaviour ←PBC | 0.226 | 0.133 | (-0.035, 0.487) | 0.090 | No |
|  | H5 | Behaviour ← Intention | 0.144 | 0.120 | (-0.090, 0.379) | 0.228 | No |
|  | H6 | Cov (Attitude, SN) | 0.387 | 241.189 | (-472.34, 473.109) | 0.999 | No |
|  | H7 | Cov (Attitude, PBC) | 0.672 | 418.784 | (-820.129, 821.473) | 0.999 | No |
|  | H8 | Cov (SN, PBC) | 0.660 | 0.083 | (0.497, 0.822) | <0.001 | Yes |
| Note: β = Standardized coefficient; SE = Standardized Error; 95%CI = 95% Confidence Interval; SN = Subjective Norm; PBC = Perceived behavioural Control. | | | | | | | |

Supplementary Table 3. Models for sensitivity analysis

| **Model** | **Classification Method of referral behaviour (times)** |
| --- | --- |
| Model 0 (Ref) | 0, 1-9, >= 10 |
| Model 1 | 0, 1-2, >= 3 |
| Model 2 | 0, 1-7, >= 8 |
| Model 3 | 0, 1-14, >= 15 |
| Model 4 | 0, 1-19, >= 20 |
| Model 5 | 0, 1-49, >= 50 |
| Model 6 | 0, 1-99, >= 100 |
| Model 7 | 0, 1-4, 5-14, >= 15 |
| Model 8 | 0, 1-9, 10-19, >= 20 |
| Model 9 | 0, 1-4, 5-9, 10-14, >= 15 |

Supplementary Table 4. Sensitivity analysis of the total model (n=330) in relation to different classifications of referral behaviour

| **Parametre** | **Estimate** | | | | | | | | | |
| --- | --- | --- | --- | --- | --- | --- | --- | --- | --- | --- |
|  | **Model 0** | **Model 1** | **Model 2** | **Model 3** | **Model 4** | **Model 5** | **Model 6** | **Model 7** | **Model 8** | **Model 9** |
| Standardized coefficients |  |  |  |  |  |  |  |  |  |  |
| Intention ← ATT | 0.021 | 0.022 | 0.021 | 0.021 | 0.021 | 0.021 | 0.021 | 0.022 | 0.021 | 0.021 |
| Intention ←SN | 0.703*** | 0.704*** | 0.703*** | 0.704*** | 0.704*** | 0.704*** | 0.704*** | 0.704*** | 0.704*** | 0.704*** |
| Intention ←PBC | 0.234** | 0.234** | 0.234** | 0.235** | 0.235** | 0.235** | 0.235** | 0.234** | 0.235** | 0.235** |
| Behaviour ←PBC | 0.140 | 0.085 | 0.152 | 0.113 | 0.090 | 0.093 | 0.065 | 0.125 | 0.133 | 0.131 |
| Behaviour ← Intention | 0.217** | 0.251*** | 0.211** | 0.243** | 0.238** | 0.203** | 0.225** | 0.235** | 0.214** | 0.226** |
| Cov (ATT, SN) | 0.444*** | 0.444*** | 0.444*** | 0.444*** | 0.444*** | 0.444*** | 0.444 | 0.444*** | 0.444*** | 0.444*** |
| Cov (ATT, PBC) | 0.662*** | 0.660*** | 0.663*** | 0.661*** | 0.660*** | 0.660*** | 0.659 | 0.661*** | 0.662*** | 0.661*** |
| Cov (SN, PBC) | 0.510*** | 0.508*** | 0.511*** | 0.508*** | 0.507*** | 0.507*** | 0.506 | 0.509 | 0.209*** | 0.509*** |
| Fit statistics |  |  |  |  |  |  |  |  |  |  |
| *χ^2^*(df) | 55.719(34) | 61.351(34) | 56.783(34) | 51.177(34) | 50.497(34) | 51.587(34) | 54.597(34) | 52.638(34) | 50.206(34) | 49.692(34) |
| RMSEA | 0.044 | 0.049 | 0.045 | 0.039 | 0.038 | 0.040 | 0.043 | 0.041 | 0.038 | 0.037 |
| ΔRMSEA | - | 0.005 | 0.001 | -0.005 | -0.006 | -0.004 | -0.001 | -0.003 | -0.006 | -0.007 |
| SRMR | 0.040 | 0.042 | 0.041 | 0.038 | 0.038 | 0.038 | 0.039 | 0.038 | 0.038 | 0.037 |
| CFI | 0.982 | 0.979 | 0.983 | 0.987 | 0.987 | 0.986 | 0.984 | 0.986 | 0.988 | 0.988 |
| ΔCFI | - | -0.003 | 0.001 | 0.005 | 0.005 | 0.004 | 0.002 | 0.004 | 0.006 | 0.006 |
| TLI | 0.973 | 0.966 | 0.972 | 0.979 | 0.979 | 0.978 | 0.974 | 0.977 | 0.980 | 0.980 |
| Note: Coefficients are standardized values. *p < 0.05, **p < 0.01, ***p < 0.001. ATT = Attitude; SN = Subjective Norm; PBC = Perceived behavioural Control; RMSEA = Root Mean Square Error of Approximation; ΔRMSEA = changes of RMSEA compared with Model 0; SRMR = Standardized root mean squared residual; CFI = Comparative Fit Index; ΔCFI = changes of CFI compared with Model 0; TLI = Tucker-Lewis Index. | | | | | | | | | | |

Supplementary Table 5. Sensitivity analysis of Group 1 model (n=162) in relation to different classifications of referral behaviour

| **Parametre** | **Estimate** | | | | | | | | | |
| --- | --- | --- | --- | --- | --- | --- | --- | --- | --- | --- |
|  | **Model 0** | **Model 1** | **Model 2** | **Model 3** | **Model 4** | **Model 5** | **Model 6** | **Model 7** | **Model 8** | **Model 9** |
| Standardized coefficients |  |  |  |  |  |  |  |  |  |  |
| Intention ← ATT | 0.087 | 0.087 | 0.087 | 0.087 | 0.087 | 0.086 | 0.086 | 0.088 | 0.088 | 0.088 |
| Intention ←SN | 0.561*** | 0.561*** | 0.561*** | 0.561*** | 0.561*** | 0.561*** | 0.561*** | 0.561*** | 0.561*** | 0.561*** |
| Intention ←PBC | 0.255** | 0.256** | 0.255** | 0.255** | 0.255** | 0.257** | 0.257** | 0.255** | 0.254** | 0.255** |
| Behaviour ←PBC | 0.100 | 0.092 | 0.097 | 0.105 | 0.096 | 0.050 | 0.049 | 0.106 | 0.106 | 0.110 |
| Behaviour ← Intention | 0.248** | 0.253** | 0.254** | 0.244** | 0.247** | 0.192* | 0.215* | 0.257** | 0.246** | 0.245** |
| Cov (ATT, SN) | 0.567*** | 0.568*** | 0.568*** | 0.568*** | 0.568*** | 0.568*** | 0.568*** | 0.568*** | 0.567*** | 0.568*** |
| Cov (ATT, PBC) | 0.617*** | 0.617*** | 0.617*** | 0.617*** | 0.617*** | 0.618*** | 0.617*** | 0.616*** | 0.617*** | 0.616*** |
| Cov (SN, PBC) | 0.474*** | 0.474*** | 0.474*** | 0.473*** | 0.473*** | 0.473*** | 0.473*** | 0.472*** | 0.473*** | 0.473*** |
| Fit statistics |  |  |  |  |  |  |  |  |  |  |
| *χ^2^*(df) | 55.829(44) | 55.854(44) | 55.879(44） | 55.953(44） | 56.733(44) | 53.144(44) | 54.472(44) | 55.695(44) | 57.654(44) | 56.555(44) |
| RMSEA | 0.041 | 0.041 | 0.041 | 0.041 | 0.042 | 0.036 | 0.038 | 0.041 | 0.044 | 0.042 |
| ΔRMSEA | - | 0.000 | 0.000 | 0.000 | 0.001 | -0.005 | -0.003 | 0.000 | 0.003 | 0.001 |
| SRMR | 0.050 | 0.049 | 0.050 | 0.049 | 0.050 | 0.048 | 0.049 | 0.049 | 0.051 | 0.049 |
| CFI | 0.984 | 0.984 | 0.984 | 0.984 | 0.983 | 0.987 | 0.986 | 0.984 | 0.981 | 0.983 |
| ΔCFI | - | 0.000 | 0.000 | 0.000 | -0.001 | 0.003 | 0.002 | 0.000 | -0.003 | -0.001 |
| TLI | 0.976 | 0.976 | 0.976 | 0.976 | 0.974 | 0.981 | 0.978 | 0.976 | 0.972 | 0.974 |
| Note: Coefficients are standardized values. *p < 0.05, **p < 0.01, ***p < 0.001. ATT = Attitude; SN = Subjective Norm; PBC = Perceived behavioural Control; RMSEA = Root Mean Square Error of Approximation; ΔRMSEA = changes of RMSEA compared with Model 0; SRMR = Standardized root mean squared residual; CFI = Comparative Fit Index; ΔCFI = changes of CFI compared with Model 0; TLI = Tucker-Lewis Index. | | | | | | | | | | |

Supplementary Table 6. Sensitivity analysis of Group 2 model (n=168) in relation to different classifications of referral behaviour

| **Parametre** | **Estimate** | | | | | | | | | |
| --- | --- | --- | --- | --- | --- | --- | --- | --- | --- | --- |
|  | **Model 0** | **Model 1** | **Model 2** | **Model 3** | **Model 4** | **Model 5** | **Model 6** | **Model 7** | **Model 8** | **Model 9** |
| Standardized coefficients |  |  |  |  |  |  |  |  |  |  |
| Intention ← ATT | -0.040 | **-** | **-** | **-** | -0.042 | -0.059 | 0.021 | **-** | -0.046 | -0.043 |
| Intention ←SN | 0.617 | **-** | **-** | **-** | 0.618*** | 0.617 | 0.630 | **-** | 0.617 | 0.619 |
| Intention ←PBC | 0.308 | **-** | **-** | **-** | 0.311 | 0.325 | 0.231 | **-** | 0.316 | 0.311 |
| Behaviour ←PBC | 0.226 | **-** | **-** | **-** | 0.139 | 0.199 | 0.215 | **-** | 0.168 | 0.184 |
| Behaviour ← Intention | 0.144 | **-** | **-** | **-** | 0.184 | 0.164 | 0.139 | **-** | 0.165 | 0.176 |
| Cov (ATT, SN) | 0.387 | **-** | **-** | **-** | 0.388 | 0.452 | 1.056 | **-** | 0.393 | 0.394 |
| Cov (ATT, PBC) | 0.672 | **-** | **-** | **-** | 0.673 | 0.781 | 1.823 | **-** | 0.683 | 0.682 |
| Cov (SN, PBC) | 0.660*** | - | - | - | 0.653*** | 0.660*** | 0.664*** | - | 0.651*** | 0.653*** |
| Fit statistics |  |  |  |  |  |  |  |  |  |  |
| *χ^2^*(df) | 10.205(0.747) | - | - | - | 10.175(14) | 11.166(14) | 16.286(14) | - | 7.563(14) | 9.327(14) |
| RMSEA | <0.001 | **-** | **-** | **-** | <0.001 | <0.001 | 0.031 | **-** | <0.001 | <0.001 |
| ΔRMSEA | - | - | - | - | 0.000 | 0.000 | 0.031 | - | 0.000 | 0.000 |
| SRMR | 0.031 | **-** | **-** | **-** | 0.031 | 0.034 | 0.040 | **-** | 0.026 | 0.029 |
| CFI | 1.000 | **-** | **-** | **-** | 1.000 | 1.000 | 0.995 | **-** | 1.000 | 1.000 |
| ΔCFI | - | - | - | - | 0.000 | 0.000 | -0.005 | - | 0.000 | 0.000 |
| TLI | 1.018 | - | - | - | 1.018 | 1.013 | 0.989 | - | 1.030 | 1.022 |
| Note: Coefficients are standardized values. *p < 0.05, **p < 0.01, ***p < 0.001. ATT = Attitude; SN = Subjective Norm; PBC = Perceived behavioural Control; RMSEA = Root Mean Square Error of Approximation; ΔRMSEA = changes of RMSEA compared with Model 0; SRMR = Standardized root mean squared residual; CFI = Comparative Fit Index; ΔCFI = changes of CFI compared with Model 0; TLI = Tucker-Lewis Index. | | | | | | | | | | |

Supplementary Table 7. Demographic and socio-economic characteristics of the permanent resident population of Yangxi County (2020）

| **Characteristics** | | **N** | **%** |
| --- | --- | --- | --- |
| Gender | Male | 230434 | 53.1 |
|  | Female | 203733 | 46.9 |
| Age, years | 0-14 | 98710 | 22.7 |
|  | 15-59 | 260681 | 60.1 |
|  | 60 and over | 74685 | 17.2 |
|  | (65 and over) | 54342 | 12.5 |
| Fertility rate (%) | | 10.33% | |
| Mortality (%) | | 3.97% | |
| Living region | Rural area | 244210 | 56.3 |
|  | Urban area | 189866 | 43.7 |
| Education years (Mean, year) | | 8.78 | |
| Education level | illiteracy | 15005 | 3.5 |
|  | Primary school | 138255 | 31.9 |
|  | Junior high school | 152369 | 35.1 |
|  | Secondary school | 55697 | 12.8 |
|  | Junior college or above | 29574 | 6.8 |
| personal consumption expenditure [Mean, CNY (USD)] ^a^ | | ¥19181 ($2789.11) | |
| personal consumption expenditure in rural areas [Mean, CNY (USD)] ^a^ | | ¥14888 ($2164.87) | |
| personal consumption expenditure in urban areas [Mean, CNY (USD)] ^a^ | | ¥22896 ($3329.31) | |
| personal disposable income [Mean, CNY (USD)] | | ¥23649 ($3438.80) | |
| personal disposable income in rural areas [Mean, CNY (USD)] | | ¥20123 ($2926.09) | |
| personal disposable income in towns and urban areas [Mean, CNY (USD)] | | ¥28334 ($4120.05) | |
| Health workers per 1000 people (Mean) | | 5.03 | |
| Licensed physicians per 1000 people (Mean) | | 1.01 | |
| Licensed physicians' assistants per 1000 people (Mean) | | 1.60 | |
| Registered nurses per 1000 people (Mean) | | 2.05 | |
| Note: ^a^ Citywide data was used. | | | |
